# 266. A Rare Case of Human Herpesvirus-6 (HHV-6) Encephalitis in an Immunocompetent Host

**DOI:** 10.1093/ofid/ofab466.468

**Published:** 2021-12-04

**Authors:** Ali M Ayyash, Muhannad Kurtom, Charlie Ervin, Mark Irwin, Rahul Sampath

**Affiliations:** 1 Carolinas Medical Center, Morganton, North Carolina; 2 Carolinas Medical Center Blue Ridge, Morganton, North Carolina; 3 Carolinas Healthcare System, Morganton, North Carolina; 4 Carolinas Health Care System Blue Ridge, Morganton, North Carolina; 5 Carolinas HealthCare Systems BlueRidge, Morganton, NC

## Abstract

**Background:**

Human Herpesvirus-6 (HHV-6) seroprevalence rates in the United States range from 72-95%, but clinical illness in the adult population is extremely rare, which often presents as meningoencephalitis in immunocompromised hosts. The literature on HHV-6 encephalitis in immunocompetent adults is limited to a select number of case reports, ultimately providing scant treatment guidance for clinicians.

**Methods:**

This is a unique case describing the clinical course of confirmed HHV-6 encephalitis in an immunocompetent host.

**Results:**

The patient is a 77-year-old immunocompetent female presenting with two days of global aphasia and increased muscle tone. She presented hypertensive with a leukocytosis. Work-up for acute stroke was unremarkable, but lumbar puncture revealed an elevated white blood cell (WBC) count of 39 leukocytes/mm^3^ with a lymphocytic predominance. BioFire FilmArray® Meningitis/Encephalitis panel (FAME) demonstrated positivity for HHV-6 with a viral load of 8,500 copies/mL in the cerebrospinal fluid (CSF) and 4.1 million copies/mL in serum. The patient experienced temporary improvement in her aphasia after being initiated on intravenous (IV) ganciclovir for 12 days. Shortly after the initiation of therapy, her aphasia worsened with repeat CSF studies demonstrating an increased viral load to 35,700 copies/mL. She was subsequently transitioned to IV foscarnet for HHV-6B coverage and discharged after completing 21 days of therapy with marked improvement in her symptoms. Two weeks later, the patient was readmitted for recurrence of aphasia. MRI brain at that time was unremarkable with repeat lumbar puncture demonstrating a WBC count of 8 with 113 copies/mL of HHV-6. Serum levels were also elevated to 4.7 million c/mL. The patient was restarted on foscarnet but continued to deteriorate clinically. She ultimately experienced multiple seizure-like episodes resulting in a noncommunicative, somnolent state. She was transitioned to hospice care and passed away 2 days after discharge.

**Conclusion:**

Despite the use of recommended medical therapies, the mortality and clinical progression of HHV-6 in immunocompetent adults is still unpredictable. Further studies are needed in this population to provide guidance for clinicians.

**Disclosures:**

**All Authors**: No reported disclosures

